# Large pyroelectric energy conversion in lead scandium tantalate thin films

**DOI:** 10.1016/j.heliyon.2024.e30430

**Published:** 2024-04-28

**Authors:** Ashwath Aravindhan, Sebastjan Glinsek, Stephanie Girod, Alfredo Blazquez Martinez, Torsten Granzow, Veronika Kovacova, Emmanuel Defay

**Affiliations:** a Materials Research and Technology Department, Luxembourg Institute of Science and Technology (LIST), 41 Rue Du Brill, L-4422 Belvaux, Luxembourg; b University of Luxembourg, 2 Avenue de L’Université, Esch-sur-Alzette L-4365, Luxembourg

## Abstract

Non-linear pyroelectric energy harvesting using ferroelectric thin films exhibits high energy conversion, primarily due to their large breakdown field compared to bulks. Here, we report the pyroelectric energy conversion potential of lead scandium tantalate, Pb(Sc_1/2_Ta_1/2_)O_3_ (PST) thin film fabricated on a c-sapphire substrate using chemical solution deposition. To enable the application of high electric field and to assess the pyroelectric energy conversion performance, interdigitated electrodes were deposited on the PST thin film. A maximum harvested energy density of 9.1 J cm^−3^ per cycle was deduced from polarization measurements in films undergoing an Olsen cycle between 0 °C and 150 °C when the electric field was varied between 50 and 1500 kV/cm. Furthermore, PST thin films can reach up to 27 % of Carnot efficiency for a temperature interval of 10 K between 30 °C and 40 °C. This study highlights the significance of PST thin films for electro-thermal energy harvesting and promising opportunities for enhancing the conversion efficiency and power density using thin films or thin film multi-layer capacitors in the future for thermal energy harvesting.

## 1 Introduction

Waste heat accounts for over 60 % of the global energy production, mostly from electricity generation [1]. Pyroelectric energy conversion (PEC) offers a potential solution to convert waste heat directly into electrical energy using temperature variations over time.

Ferroelectric materials are favoured for PEC due to their strong dependency of polarization on temperature and electric field. Olsen et al. [2,3] demonstrated non-linear pyroelectric energy harvesting by exploiting the field-induced transition of bulk ferroelectric ceramics. Additionally, by implementing heat regeneration, they obtained more than 6 J of electrical energy using Sn-doped Pb(Zr,Ti)O_3_ bulk ceramics in a macroscopic pyroelectric energy harvester [4]. Recently, it was reported that more than 10 J of energy per cycle can be generated using Pb(Sc_1/2_Ta_1/2_)O_3_ (PST) multi-layer capacitors in a macroscopic prototype [5]. Moreover, PST multi-layer capacitors can generate up to 4.4 J cm^−3^ of energy density per cycle [5].

The pyroelectric energy density scales with the applied electric field; thin films offer the possibility to enhance energy density as they afford increased dielectric strength [6]. In a previous study, Sebald et al. [7] reported that it is even possible to achieve up to 50 % of Carnot efficiency using thin films, as higher energy densities could be attained using thin films. However, directly measuring PEC and electrocaloric effect (converse of pyroelectric effect) in thin films is challenging due to their small volume and thermal mass [8,9], necessitating indirect estimation methods.

Earlier reports showed doped Pb(Zr,Ti)O_3_ thin and thick films achieving over 7 J cm^−3^ of pyroelectric energy density per cycle [10,11]. Recently, Bhatia et al. [12] and Pandya et al. [13,14] revived interest in pyroelectric thin films by implementing PEC cycles on them and directly measuring their energy and power density. Thin films offer the advantage of faster cycling compared to bulk ceramics as the heat exchange is rapid due to its small thermal time constant. Zhang et al. [15] demonstrated the potential of thin films by recharging the battery through PEC, at frequencies as high as 1 kHz. Besides, thin films could also be easily integrated into microelectronic devices for thermal energy harvesting as considerable energy is lost in the form of low-grade waste heat. Using thin-film multilayer capacitors could be a viable solution for PEC and further miniaturization of integrated components in a device.

PST is an exceptional material for thermal energy interconversion due to its peculiar phase transition behaviour close to room temperature. This makes PST an ideal candidate for harvesting energy from common ambient temperature variations as significant energy output is obtained near the transition. PST transition behaviour can be altered from a relaxor to a conventional ferroelectric material (sharp first-order transition) by alternative arrangement of Sc^3+^ and Ta^5+^ ions on B-site of its perovskite structure (rock-salt ordering) through annealing [16]. Both chemically ordered (first order transition) and disordered (relaxor) PST structures are interesting for electrocaloric and pyroelectric energy harvesting applications. Recently, it was suggested that a large number of possible polar states could be obtained in a relaxor ferroelectric by defect modification, to yield a larger electrocaloric effect [17,18]. On the other hand, high pyroelectric coefficients can be obtained in a chemically homogeneous (i.e., B-site ordered) PST, leading to large electrocaloric and PEC effect [19]. Extensive research has also been carried out towards the synthesis and optimization of PST bulk ceramics and thin films [[20], [21], [22], [23]]. However, the literature on PST thin films for electro-thermal energy conversion is rather limited [24,25] and there is no available report on non-linear pyroelectric energy harvesting.

In this study, we report the PEC potential of PST thin films on a sapphire substrate with interdigitated top electrodes as the traditional metal–insulator–metal configuration imposes constraints on the processing of PST films. Deducing the possible energy output of an Olsen cycle from polarization measurements, we were able to obtain a maximum energy density of 9.1 J cm^−3^ per cycle for a ΔT of 150 K and ΔE of 1450 kV/cm. Moreover, the relative efficiency of PST thin film could reach up to 27 % Carnot efficiency for a 10 K temperature span.

## 2 Methods

Pb(Sc_1/2_Ta_1/2_)O_3_ solution with 30 % excess Pb was synthesized using lead (Ⅱ) acetate trihydrate, scandium (Ⅲ) acetate hydrate, tantalum (Ⅴ) ethoxide metal precursors, and 2-methoxyethanol solvent. Reagents for PST processing (>99.9 % purity) were obtained from *Merck, Germany*. Lead (Ⅱ) acetate trihydrate and scandium (Ⅲ) acetate hydrate precursors were freeze dried for 48 h to remove the water. Then, scandium (Ⅲ) acetate and tantalum (Ⅴ) ethoxide precursors were dissolved in 2-methoxyethanol and were fluxed for 24 h under argon atmosphere. An additional reflux step for 2 h was done after introducing lead (Ⅱ) acetate precursor to the mixture, followed by a distillation to remove the by-products. The resulting solution was diluted with 2-methoxyethanol to achieve a final molar concentration of 0.3 M.

PST thin films were grown on c-sapphire substrates (Siegert Wafer). The substrate was first coated with a 23 nm thick hafnia buffer layer using atomic layer deposition [26]. To initiate the desired perovskite structure, lead titanate (0.1 M concentration) was used as a seed layer using sol-gel deposition [27] with the conditions reported in the literature [28]. PST solution was then spin coated at 3000 rpm for 30 s followed by drying and pyrolysis for 120 s at 130 °C and 350 °C respectively. These steps were successively repeated in sequence four times to yield a film thickness of 165 ± 5 nm (see Supplementary Material Fig.S1). PST crystallization was carried out in a rapid thermal annealing furnace under air atmosphere at 800 °C for 15 min with a heating rate of 50 °C/s and it was cooled naturally in the furnace.

**Fig. 1:**
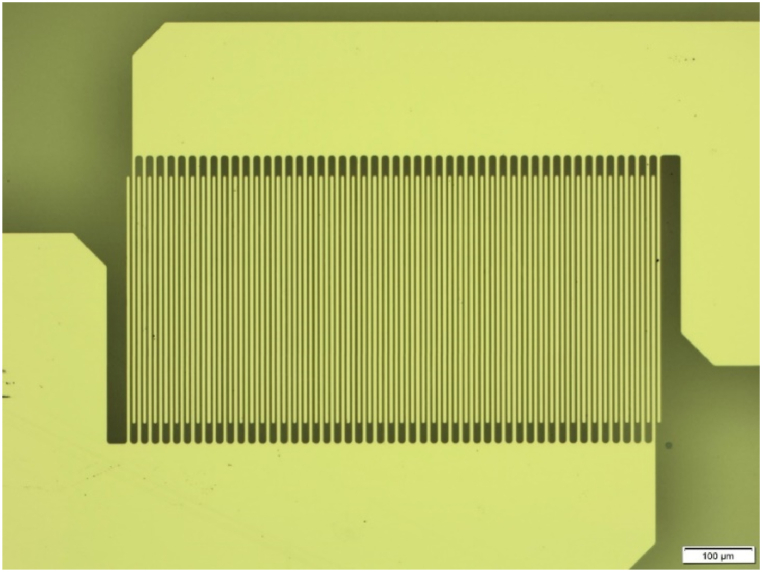
Optical microscope image of the IDE geometry.

Interdigitated electrodes were patterned on top of the PST thin film using conventional lift-off photolithography (MLA 150, Heidelberg instruments), and 100 nm-thick platinum electrodes were deposited using sputtering. The IDE pattern consists of a total of 50 pairs of fingers, with an effective length of 370 μm, a finger width of 5 μm, and a spacing of 3 μm between the fingers (see Fig. 1). To prevent electrical arcs between the fingers under high voltage, a 2 μm thick SU-8 photoresist was deposited on top of the patterned electrodes [26].

Structural characterizations were performed by x-ray diffraction (XRD) with a Bruker D8 diffractometer using Cu-K_α_ radiation in reflection geometry (*θ - 2θ configuration*). The scans were performed from 10° to 70° with a step size of 0.02° and a time interval of 2 s per step. XRD peaks were indexed using the PDF file number 01-074-2635 [29,39].

Due to the (100) preferential orientation of PST, an additional measurement in a skewed geometry was also performed to investigate the presence of B-site order. Here, the sample was tilted to an angle of *χ* = 54.7° (angle between (111) and (200) plane) and a *θ - 2θ* scan was performed with a 1 mm collimated beam between 16° and 21° with a time step of 20 s. This measurement was carried out to detect the presence of the (111) peak, which is one of the superstructure peaks that reflects the degree of chemical order in the PST film. The chemical order of an A (B_0.5_’,B_0.5_″)O_3_ perovskite material, such as PST, is found by comparing the intensity of a superstructure peak to that of a regular peak [25]. The order can be calculated as follows [16]:

$${S_{B}^{2}}_{\left(111\right)}=\frac{\left(\frac{I_{111}}{I_{222}}\right)film}{\left(\frac{I_{111}}{I_{222}}\right)ordered}$$

A detailed description of this adapted approach for estimating the chemical order in A (B_0.5_’,B_0.5_″)O_3_ perovskite thin films can be found elsewhere [25,30].

Electrical characterizations were performed using TF Analyzer 2000 (aixACCT, Germany). Bipolar polarization hysteresis loops were measured at 100 Hz at different temperatures and electric fields using a triangular waveform. Small-signal capacitance versus voltage (CV) measurements were carried out between -3V and +3V at a frequency of 1 kHz and 250 mV AC field amplitude, over a temperature range of 0–100 °C. The relative permittivity values are calculated from capacitance values at 1 kHz and zero volts. Frequency dependent hysteresis and permittivity measurements at different temperatures can be found in the Supplementary Material (fig.S3)

**Fig. 2:**
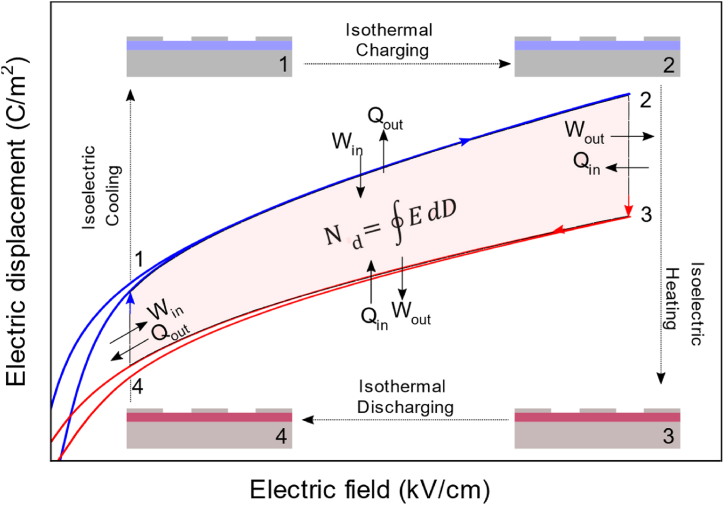
Illustration of Olsen cycle on an electric displacement versus electric field. The four steps of the cycles correspond to Refs. [1,2] charging the sample at low temperature [2,3], heating the sample at constant electric field [3,4] discharging the sample at high temperature [[1], [2], [3], [4]], cooling at low electric field to go back to the initial electric displacement value. The shaded area represents the energy density (N_d_).

Pyroelectric conversion performance was indirectly estimated using Olsen cycle from electric displacement versus field loops. An Olsen cycle (or electrical Ericsson cycle) consists of two isothermal steps and two isoelectric steps. The sample is charged at isothermal conditions and then heated while keeping the electric field constant. After reaching the high temperature, the sample is discharged isothermally followed by cooling of the sample. Fig. 2 shows the schematic of the Olsen cycle. The harvested energy can be calculated using:

(1) Nd=∮EⅆD

where *E* is the electric field and *D* is the electric displacement. The thermodynamic conversion efficiency can be estimated using the formula:

(2) $$\eta =Harvestable\;energy\;\left(N_{d}\right)/Heat\;input\;\left(Q_{in}=C.\rho .\left(\Delta T+\Delta T_{EC}\right)\right)$$

where *C* is the specific heat, ρ is the density, ΔT is the temperature span and *ΔT*_*EC*_ corresponds to the temperature variation due to the electrocaloric effect. Here, the electrocaloric effect was also considered to ensure the upper bound value for the heat input. The values of C and ρ are 300 J kg^−1^ K^−1^ and 9070 kg m^−3^ respectively [25]. The efficiency relative to Carnot can be estimated as follows:

(3) $$\eta r=\frac{\eta }{\eta_{Carnot}}=\frac{N_{d\;}T_{hot\;}}{Q_{in\;}\Delta T\;}$$

## 3 Results and discussion

**Fig. 3:**
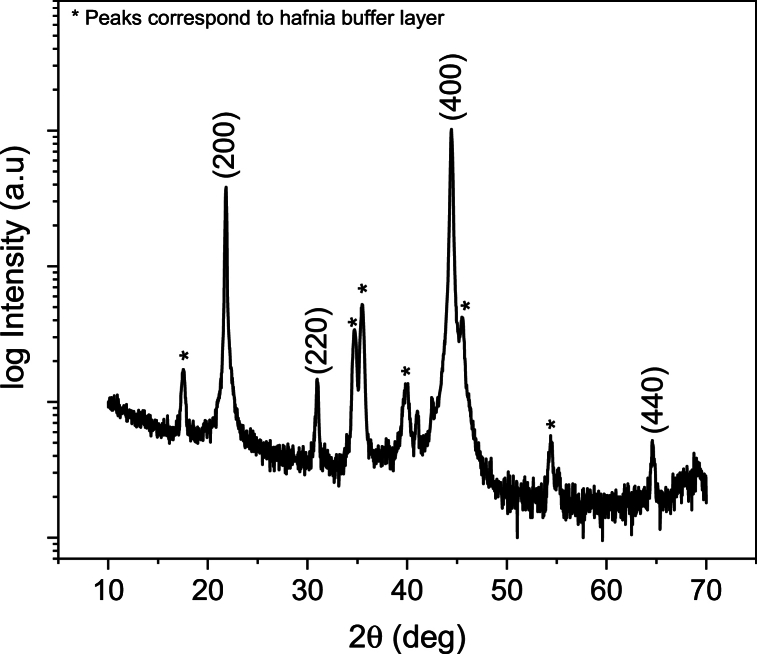
XRD pattern of the PST thin film on a c-sapphire substrate. The peaks of the perovskite phase are marked with Miller indices and stars denote the hafnia peaks.

XRD analysis (shown in Fig. 3.) confirms the presence of a single-phase perovskite structure with a strong (200) orientation with respect to powder, despite the presence of a weak (220) peak as well (note the log scale on the y-axis) [PDF card 01-074-2635] [39]. Additional peaks correspond to the dielectric hafnia buffer layer [PDF card 00-034-0104] [40]. Due to the preferential orientation of the PST thin film, the estimation of B-site chemical order from the *θ – 2θ* scan is not possible. The existence of B-site chemical order is indicated by the presence of superstructure peaks [30]. To detect the presence of (111) superstructure peak corresponding to the main texture (200) of the film, a *θ – 2θ* scan in the out-of-plane direction of (111) peak was performed by tilting the sample to 54.7°, where maximum intensity of (111) peak should be observed. However, the absence of the (111) superstructure peak at 18.87° implies the non-existence of B-site chemical ordering in PST film. The measurement details of this experiment are provided in the Methods section. Data concerning the skew geometry measurements can be found in the Supplementary Material Fig.S2.

**Fig. 4:**
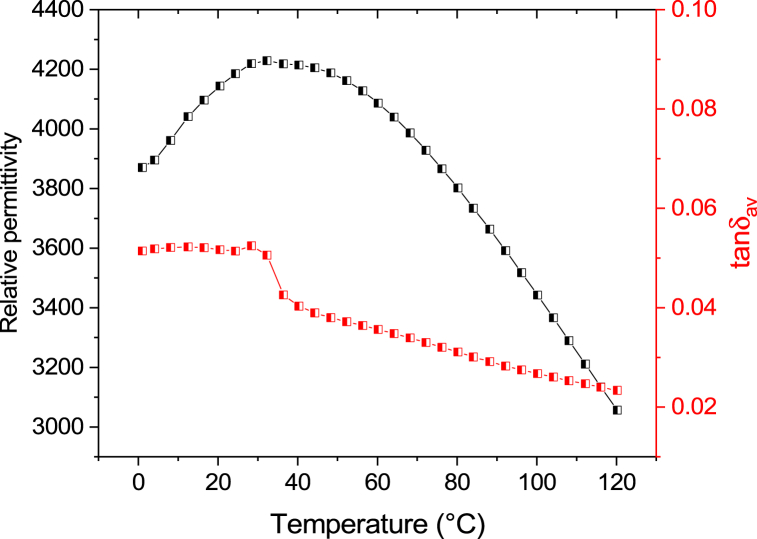
Temperature dependence of zero field permittivity and loss tangent of PST thin film at 1 kHz.

Fig. 4. shows the temperature dependence of zero-field permittivity and loss tangent of the PST film at 1 kHz. The permittivity reaches a maximum of 4230 at 32 °C. Similar values were reported for completely disordered PST thin films on c-sapphire substrate at 1 kHz in the literature [22]. Based on the broad peak of the permittivity and loss tangent at 30 °C and the clear frequency dispersion behaviour (peak position in temperature increasing with frequency, as observed in Supplementary Material Fig. S3), we could confirm the relaxor nature of the PST thin film. The existence of B-site ordering could not be detected using XRD. In a previous study, Brinkmann et al. [31] investigated the dielectric response of both disordered and ordered PST thin films. They concluded that the permittivity values increase with an increase in chemical order, in contrast to PST bulk ceramics, where the dielectric constant decreases with the increase in order. Detailed frequency dependent measurement of permittivity as a function of temperature can be found in the Supplementary Material Fig. S3.

**Fig. 5:**
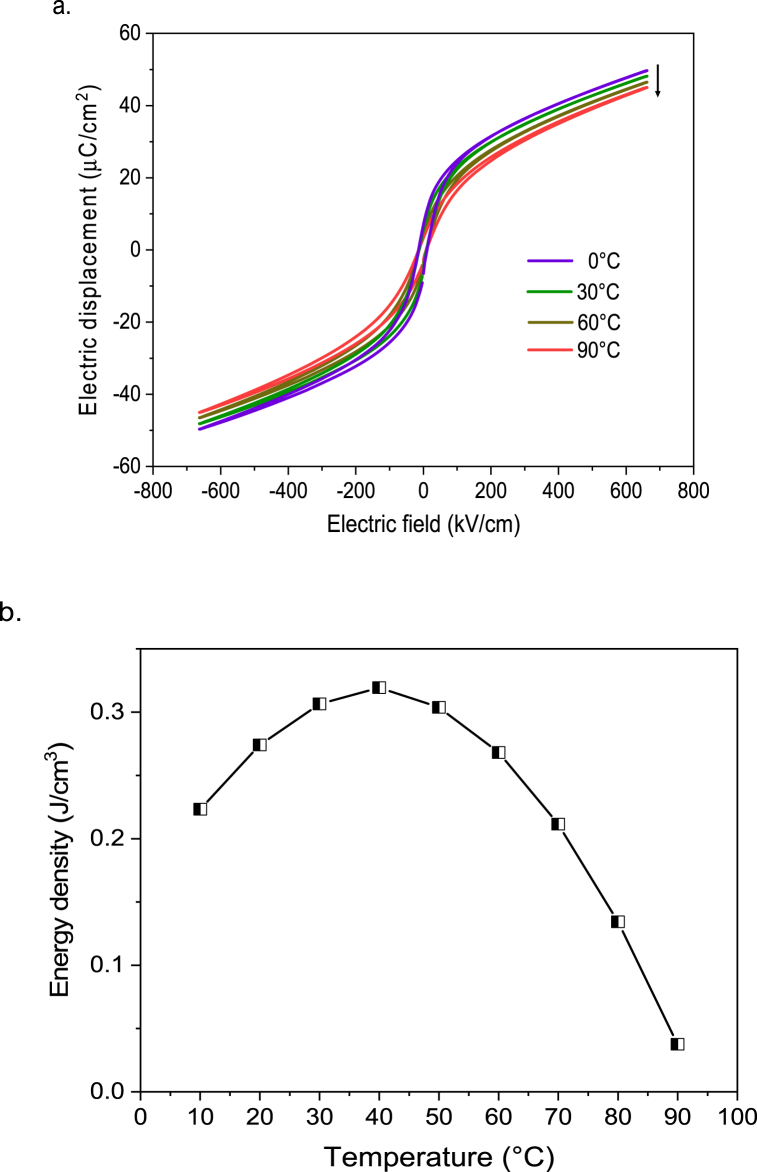
(a) Electric displacement *D* versus electric field *E* loops of PST thin film at 100 Hz from 0 to 90 °C at a maximum field of 660 kV/cm. (b) Energy density *N*_*d*_ from the *D – E* loops at every 10 °C temperature interval for a *ΔE* of 520 kV/cm.

To estimate the efficiency of PST thin film using Olsen cycle, *D – E* loops were measured at different temperatures for a field amplitude of 660 kV/cm at 100 Hz as shown in Fig. 5 a. The pyroelectric energy density values were calculated using equation (1) from the *D – E* loops for different temperature intervals. Fig. 5 b. illustrates the energy density values extracted from the *D – E* loops using Olsen cycle for a *ΔE* of 520 kV/cm (140–660 kV/cm) at every 10 °C temperature interval (the temperature on the x-axis correspond to the final temperature or step [3,4] of the Olsen cycle). A peak in the energy density (0.32 J cm^−3^ for a ΔT of 10 K) is observed when the Olsen cycle is implemented between 30 and 40 °C, which encompasses the peak of the relative permittivity.

**Fig. 6:**
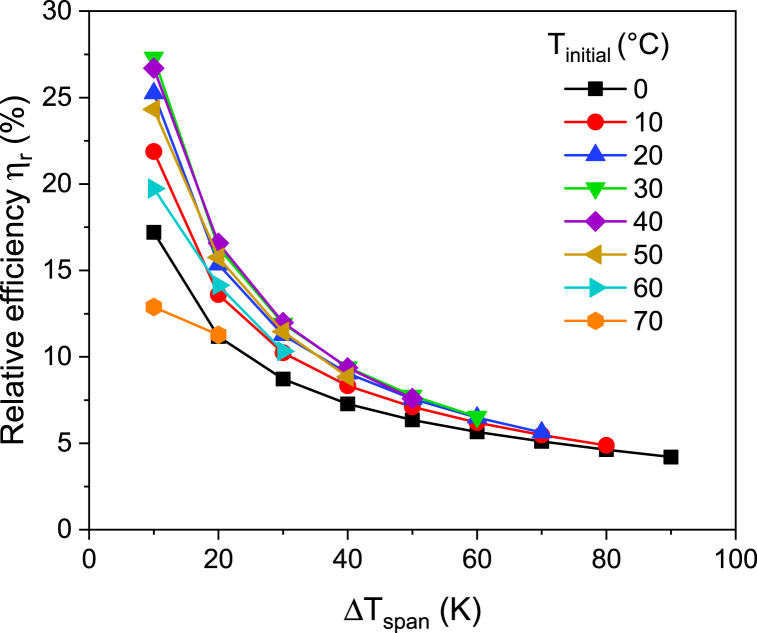
Relative efficiency of PST thin film as a function of temperature span for a *ΔE* of 520 kV/cm at different initial temperatures.

To estimate the pyroelectric conversion efficiency using equation (2), it is also necessary to take the electrocaloric effect into consideration. Here, we took the maximum electrocaloric temperature variation of 3.5 K for a *ΔE* of 520 kV/cm, which was calculated indirectly using the thermodynamic Maxwell's relation, irrespective of the operating temperature window to ensure an upper bound for the heat input. The relative efficiency is estimated from equation (3). Fig. 6 shows the relative efficiency of the PST thin film as a function of temperature span. A maximum of 27 % Carnot efficiency was observed for a 10 K temperature span at 30 °C. The relative efficiency decreased with the increase of temperature window. To obtain higher conversion efficiency in a pyroelectric material, a strong first order transition over a small temperature span is preferred. To further enhance the relative efficiency and to increase the harvesting temperature window, Olsen et al. [32] proposed to use several pyroelectric materials with sharp transition and varying transition temperatures as a cascaded device. In the case of PST ceramics, it was reported that the transition temperatures could be varied *via* doping while maintaining a sharp first order transition [33].

**Fig. 7:**
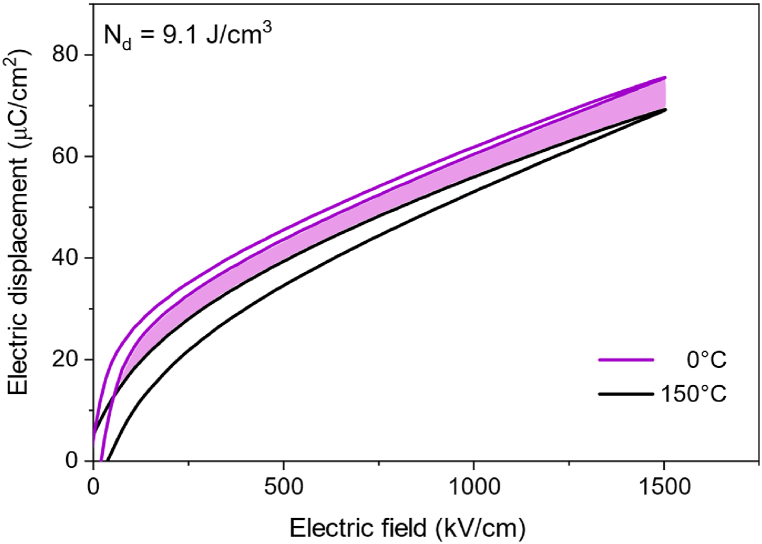
Indirect estimation of pyroelectric energy density using Olsen cycle from the *D-E* loops (enlarged) between 0 and 150 °C from 50 to 1500 kV/cm.

To investigate the maximum electrical work output of PST thin film, the *D-E* loops were measured at different temperatures, frequencies, and electric fields (see Supplementary Material Fig.S4 – S6). As the temperature span was expanded, the electrical work output also increased. A maximum energy density of 9.1 J cm^−3^ per cycle was obtained using the Olsen cycle between 0 and 150 °C when the electric field was varied between 50 and 1500 kV/cm as shown in Fig. 7. The shaded area corresponds to the harvestable energy density of PST thin film. This value is more than twice that of PST multi-layer capacitors in the literature [5] owing to their large breakdown field. Table 1 compares the pyroelectric energy harvesting potential of PST thin films and other ferroelectric materials. While the indirect measurements from the *D-E* loops offer a reliable estimate of the electrical work output as reported in our previous study [5], the preferred approach should be direct implementation and measurement of the electrical work output. In practice the electro-thermal energy conversion cycle must be optimized to minimize hysteresis losses at elevated temperatures and electric fields to achieve high electrical work output.

**Table 1:** A comparison of pyroelectric energy harvesting capability of PST thin films and other ferroelectric materials using Olsen cycle.

Material	Type –Thickness (μm)	ΔT (K)	T_i_ (°C)	Energy density (J/cm^3^)	ΔE (kV/cm)	Ref.
BaTiO_3_	Thin film −0.1	100	20	0.01	25	[12]
Pb_0.99_Nb_0.02_(Zr_0.68_Sn_0.25_Ti_0.07_)_0.98_O_3_	Ceramic −250	33	145	0.13	20	[34]
0.90 Pb(Mg_1/3_Nb_2/3_)O_3_–0.10PbTiO_3_	Ceramic −1000	50	35	0.18	30	[7]
Pb(Zn_1/3_ Nb_2/3_)_0.955_Ti_0.045_O_3_	Single crystal −1000	60	100	0.24	20	[35]
60PVDF-40TrFE	Thick film – 50	75	25	0.52	500	[36]
8/65/35 (Pb, La) (Zr,Ti)O_3_	Thick film – 290	135	25	0.88	73	[37]
(Pb_0.86_La_0.02_Ba_0.11_) (Zr_0.58_Sn_0.29_Ti_0.13_)O_3_	Ceramic −500	122	28	0.89	55	[38]
0.68 Pb(Mg_1/3_,Nb_2/3_) −0.32PbTiO_3_	Thin film −0.15	90	25	1.06	267	[14]
Pb(Sc_0.5_,Ta_0.5_)O_3_	MLC – 9 layers of 38 μm each	175	5	4.43	195	[5]
Pb_0.99_ Nb_0.02_(Zr_0.55_Sn_0.40_Ti_0.05_)_0.98_O_3_	Thin film −0.5	200	25	7.3	873	[10]
Pb_0.97_La_0.02_(Zr_0.75_Sn_0.18_Ti_0.07_)O_3_	Thick film – 2	260	25	7.8	600	[11]
Pb(Sc_0.5_,Ta_0.5_)O_3_	Thin film −0.16	150	0	9.1	1450	This work

## 4 Conclusions

This work reports the pyroelectric energy conversion potential of PST thin films fabricated on a c-sapphire substrate using sol-gel deposition technique. High breakdown field in PST films were achieved using interdigitated electrodes. Structural and electrical characterizations confirmed the relaxor nature of PST thin films. From the *D-E* loops, we estimate a large pyroelectric energy density of 9.1 J cm^−3^ per cycle for a *ΔT* of 150 K and *ΔE* of 1450 kV/cm using Olsen cycle. Furthermore, a maximum of 27 % Carnot efficiency could be achieved in PST films for a *ΔT* of 10 K and *ΔE* of 520 kV/cm. This work highlights the potential of PST thin films for pyroelectric energy harvesting and these results encourage to revisit the concept of pyroelectric energy harvesting for practical applications. In the future, it will be intriguing to investigate the pyroelectric power density and efficiency of miniaturized multilayer capacitors.

## Data availability statement

Data included in article/supplementary material/referenced in article.

## CRediT authorship contribution statement

**Ashwath Aravindhan:** Writing – original draft, Visualization, Validation, Investigation, Formal analysis, Data curation. **Sebastjan Glinsek:** Writing – review & editing, Validation, Investigation. **Stephanie Girod:** Investigation. **Alfredo Blazquez Martinez:** Software, Investigation. **Torsten Granzow:** Writing – review & editing, Validation, Investigation. **Veronika Kovacova:** Writing – review & editing, Supervision, Project administration, Methodology, Conceptualization. **Emmanuel Defay:** Writing – review & editing, Supervision, Project administration, Methodology, Funding acquisition, Conceptualization.

## Declaration of competing interest

The authors declare that they have no known competing financial interests or personal relationships that could have appeared to influence the work reported in this paper.
