# Supplementary material for: Large pyroelectric energy conversion in lead scandium tantalate thin films
Source: Heliyon. 2024 Apr 28;10(9):e30430. doi: 10.1016/j.heliyon.2024.e30430 (PMC11079096; doi:10.1016/j.heliyon.2024.e30430)
Supplement: Multimedia component 1 [file mmc1.docx]

**Appendix A. Supplementary data**

**Large pyroelectric energy conversion in lead scandium tantalate thin films**

Ashwath Aravindhan ^a,b*^ , Sebastjan Glinsek ^a^, Stéphanie Girod ^a^, Alfredo Blázquez Martínez ^a,b^, Torsten Granzow ^a^, Veronika Kovacova ^a*^, Emmanuel Defay ^a*^

^a^ Materials Research and Technology Department, Luxembourg Institute of Science and Technology (LIST), 41 Rue du Brill, L-4422 Belvaux, Luxembourg

^b^ University of Luxembourg, 2 Avenue de l’Université, Esch-sur-Alzette L-4365, Luxembourg

**Microstructure of PST thin films**


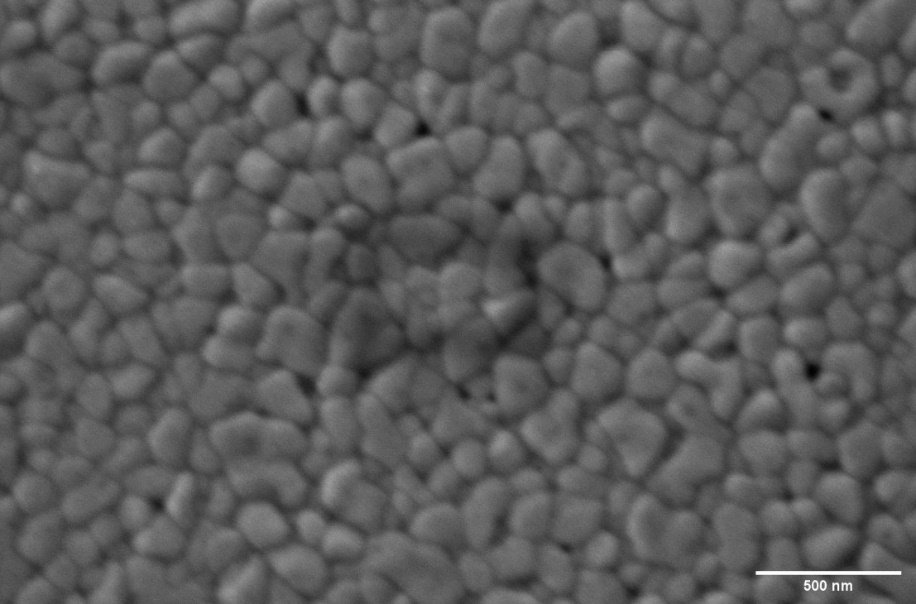


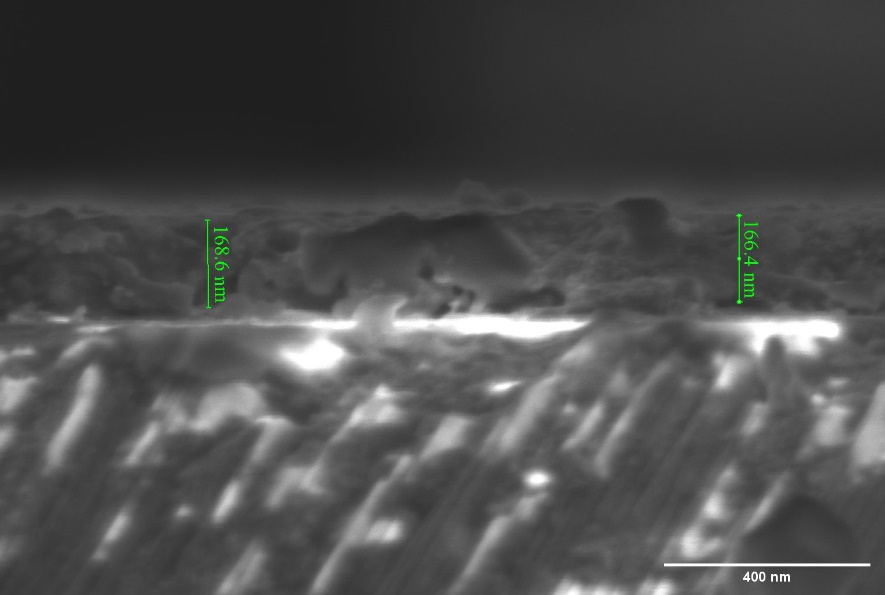


Fig.S.1. FESEM images of surface and cross-section of a PST thin film on c-sapphire substrate.

**Skew geometry XRD measurement**

To determine the presence of superstructure peak in the PST film, a *θ - 2θ* scan was performed in the out-of-plane direction of (111) peak by tilting the sample to an angle of *χ* = 54.7°. Here, the *θ - 2θ* scan was carried out from 16 to 21° with a step size of 0.02° and a time interval of 20 s per step. Fig.S.1. show the XRD measurement of PST thin film at *χ* = 54.7°. The absence of (111) peak at 18.86° [from the PDF file number 01-074-2635] confirms the non-existence of B-site cation ordering in this thin film.

Fig. S.2. XRD measurement of PST thin film at *χ* = 54.7°.

**Frequency dependent dielectric measurements**

Temperature dependent dielectric permittivity measurements were performed at different frequencies (1 kHz to 1 MHz) using Novocontrol Concept 40 dielectric spectrometer system. Initially, the bias voltage was set to a constant value and the permittivity and loss tangent of PST thin film were measured continuously at different frequencies from -50°C to 120°C. After the measurement was completed, the temperature returned to its initial value to repeat the measurement with a different bias voltage and the measurement sequence is repeated. These results affirm the relaxor nature of PST thin film (see Fig. S.2)


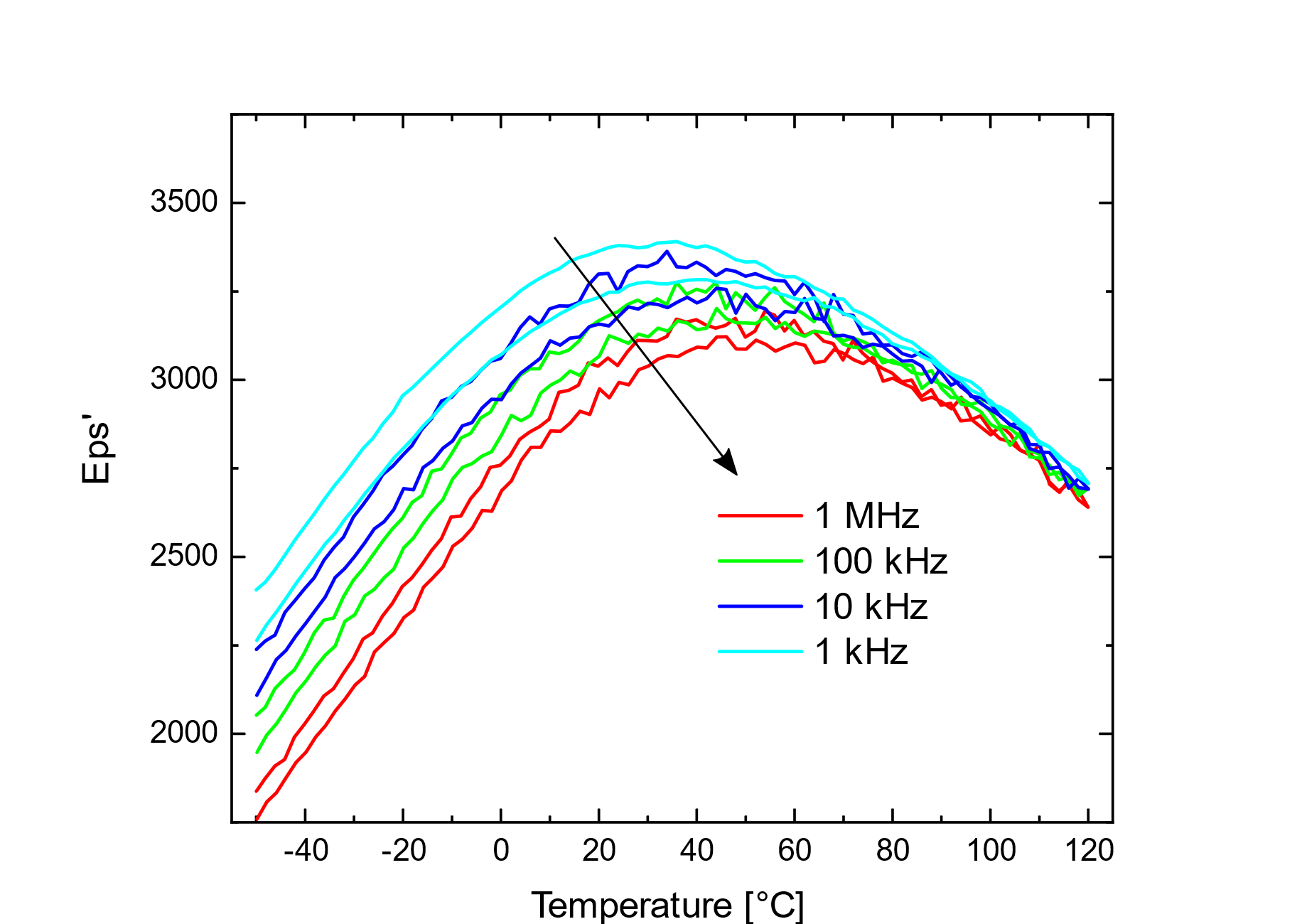


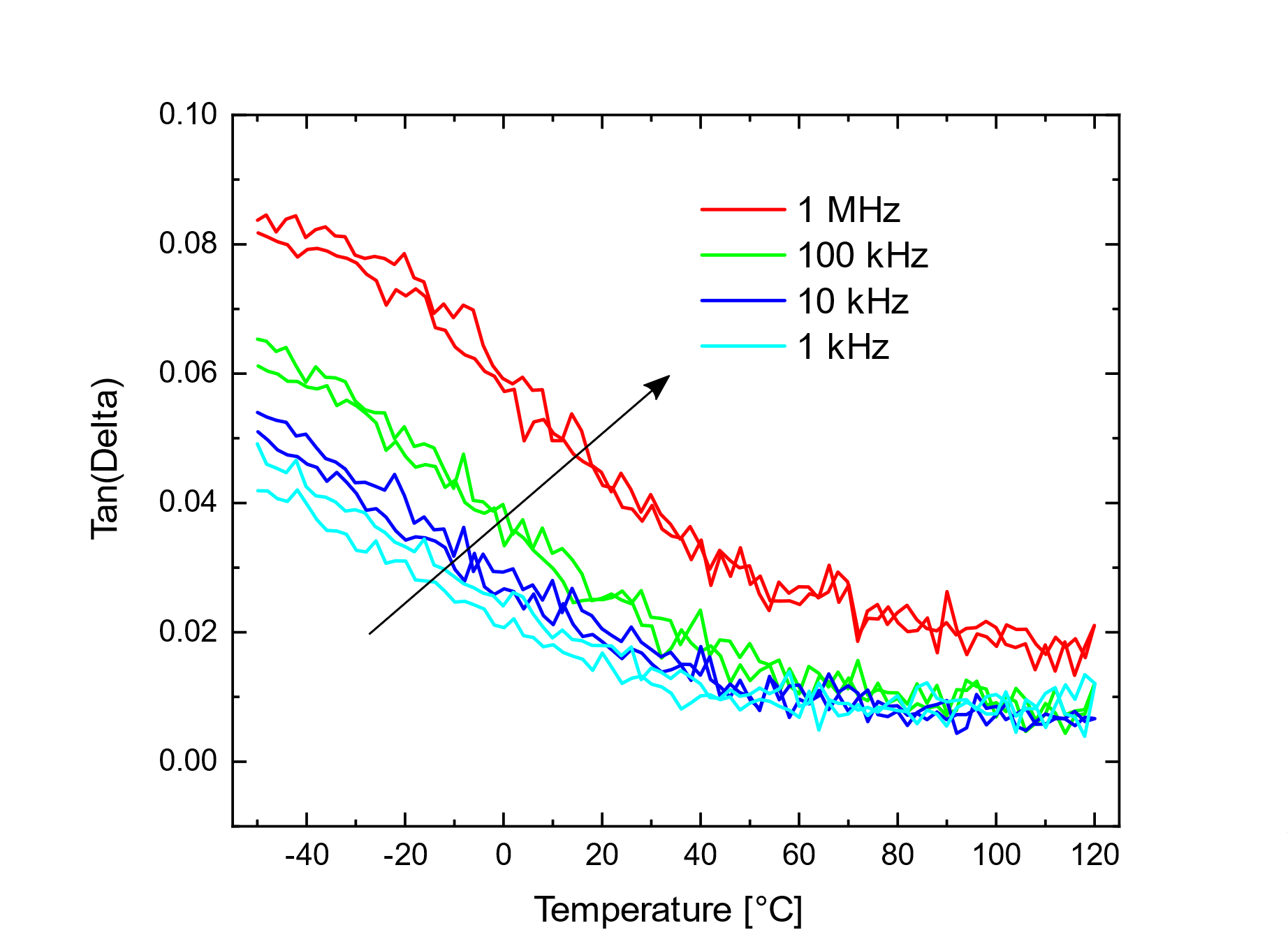


Fig. S.3. Temperature dependence of dielectric constant (top) and loss tangent (bottom) of PST thin film at different frequencies at a bias voltage of 3 V

**Electric displacement *D* versus electric field *E* loops**

To estimate the pyroelectric energy density, *D-E* loops were measured at different temperatures, electric fields, and frequencies. At room temperature, the breakdown field of PST thin film exceeded 2000 kV/cm. The high breakdown field of the PST thin film is attributed to the utilization of interdigitated electrodes.

Fig. S.4. *Electric displacement versus electric field and current density versus electric field* loop of PST thin film.

Most of the capacitors failed to survive higher electric fields at high temperatures. Though some capacitors were able to withstand fields higher than 1500 kV/cm at elevated temperatures, the pyroelectric energy density was insignificant due to the hysteresis.

Fig. S.5. *D-E loops of PST thin film at 100 Hz* (top) 1 kHz (bottom)

**Estimating the pyroelectric energy density from the *D-E* loops**

To attain higher pyroelectric energy density, the operating temperature, electric field, and the frequency must be optimized with respect to the considered electro-thermodynamic cycle. In this work, we consider only the Olsen (or pyroelectric Ericsson cycle) as it yields the highest electrical work output [1][2].

The highest energy density of 9.1 J cm^-3^ was obtained at a frequency of 100 Hz for a ΔE of 1450 kV/cm and ΔT of 150 K (main text). To verify the reproducibility of these results, similar measurements with the same parameters were carried out in a separate capacitors, resulting in a maximum energy density of 8.6 J∙cm^-3^.


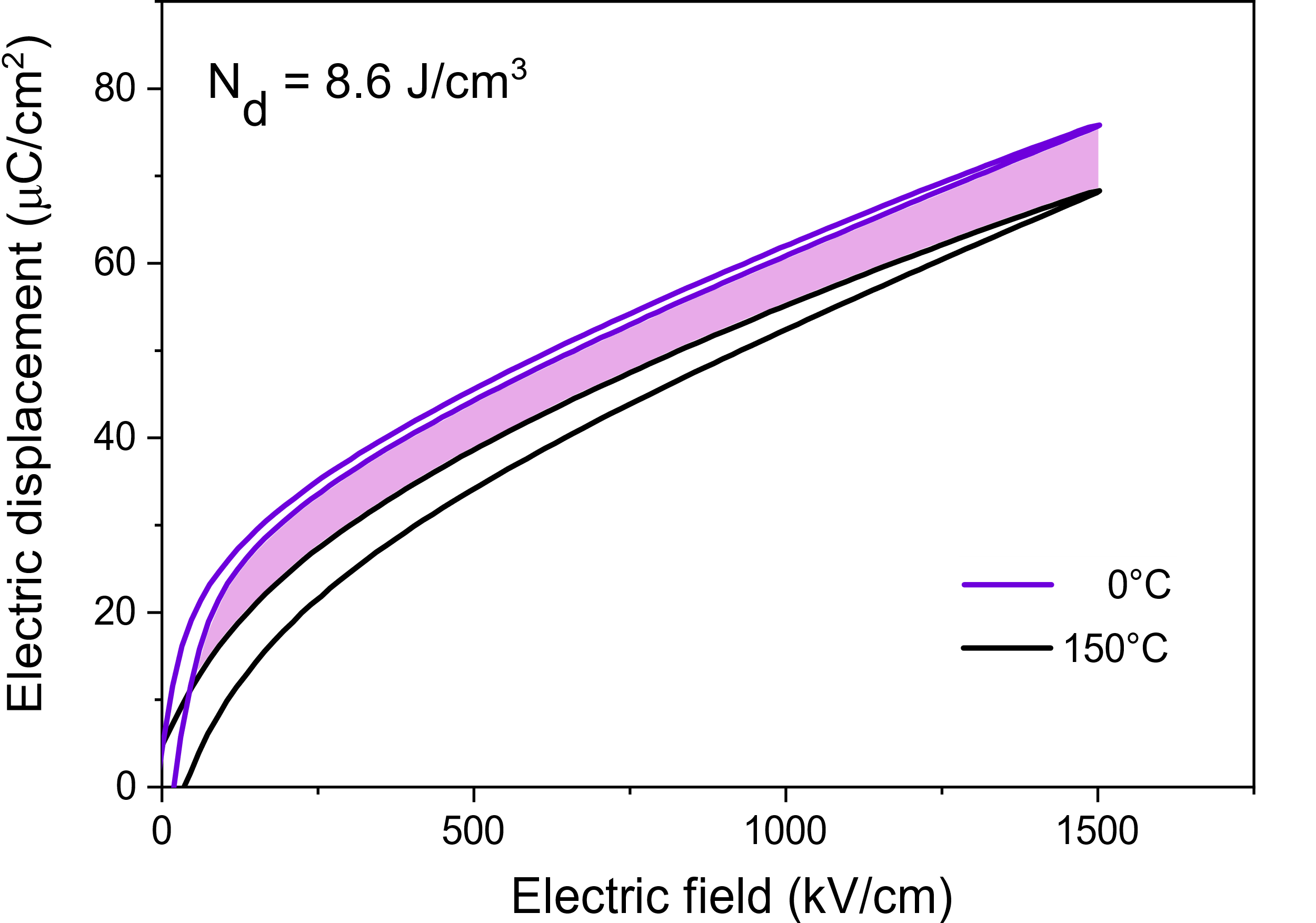


Fig. S.6. Indirect estimation of pyroelectric energy density using Olsen cycle from the *D-E* loops (enlarged) at 100 Hz between 0 and 150°C from 50 to 1500 kV/cm.

**References**

[1] Olsen RB, Brown DD. High efficiency direct conversion of pyroelectric measurements heat to electrical energy-related. Ferroelectrics 1982;40:17–27. https://doi.org/10.1080/00150198208210592.

[2] Sebald G, Pruvost S, Guyomar D. Energy harvesting based on Ericsson pyroelectric cycles in a relaxor ferroelectric ceramic. Smart Mater Struct 2008;17. https://doi.org/10.1088/0964-1726/17/01/015012.
